# A novel genotype of MVA that efficiently replicates in single cell suspensions

**DOI:** 10.1186/1753-6561-7-S6-O1

**Published:** 2013-12-04

**Authors:** Ingo Jordan, Volker Sandig

**Affiliations:** 1 ProBioGen AG, 13086 Berlin, Germany

## Background

Vectored vaccines based on modified vaccinia Ankara (MVA) may lead to new treatment options against infectious diseases and certain cancers. MVA is highly attenuated and requires avian cells for production. We established avian continuous cell lines (including CR and related CR.pIX) and adapted these cells to proliferation in single-cell suspension in a chemically defined medium [1,2]. Replication of several viruses was efficient in CR suspension cultures [3,4] but yields for MVA were low. We suspected that cell-to-cell spread may be an important mechanism for MVA replication in agitated suspension cultures and developed a production medium that is added at the time of infection to induce cell aggregates [2]. MVA (and other host-restricted poxviruses) replicate to very high titers with this robust and fully scalable cultivation protocol but further improvement may facilitate production for large vaccine programs. We now describe a novel genotype of MVA that replicates with high efficiency in single-cell suspensions without aggregate induction.

## Materials and methods

**Figure 1:**
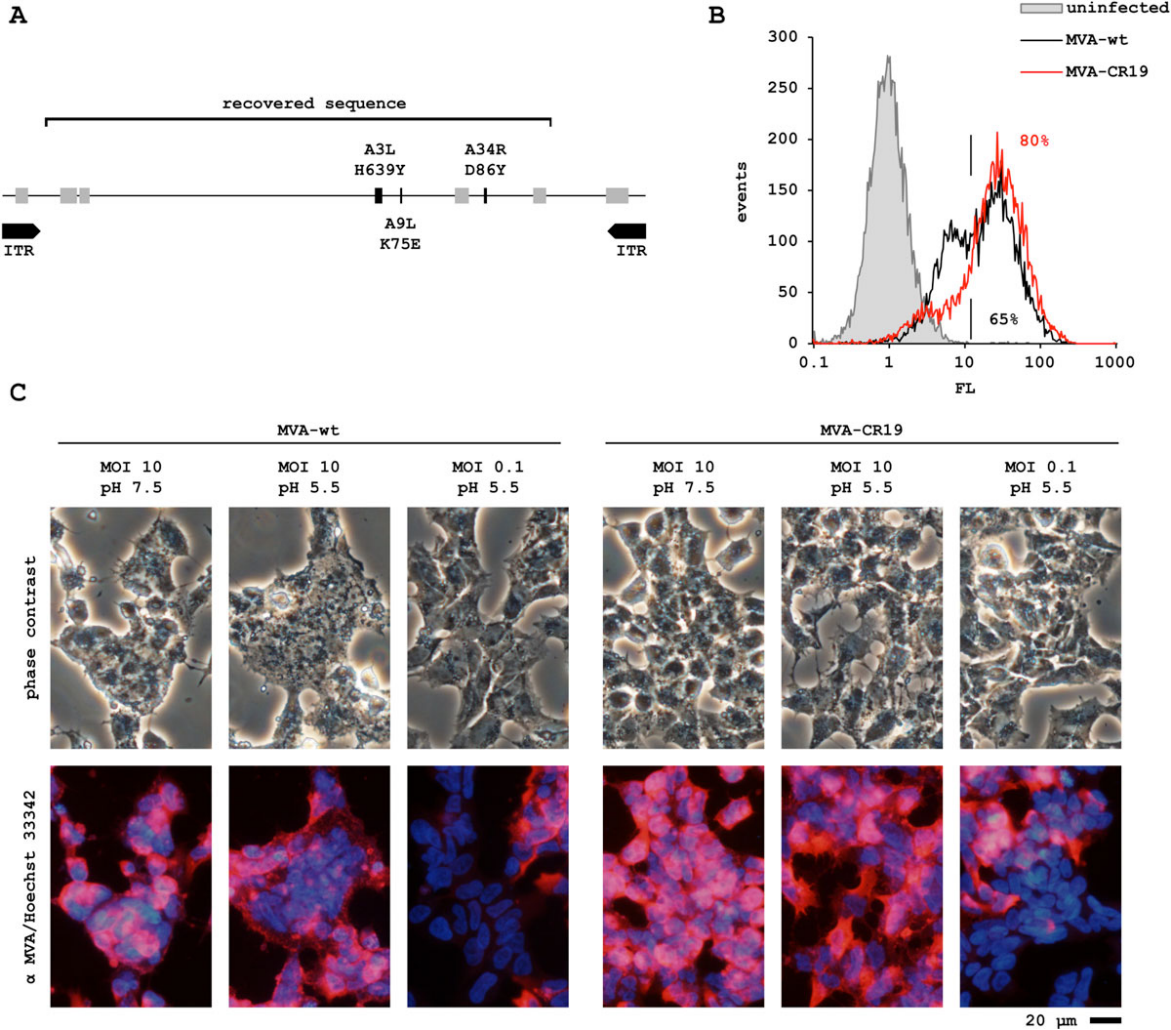
**(A) Schematic of the genomic DNA of MVA-CR**. The region covered by next generation sequencing is shown together with the mutations (in single letter code for amino acids, e.g. H639Y is His_639 _→ Tyr) in the three genes. ITR (viral telomers) and deletion sites in MVA as light gray boxes are shown for orientation. **(B) **CR.pIX single-cell suspension cultures were infected with wildtype (wt) and MVA-CR19. Cells were immunostained for virus antigens 48 h post infection and quantified by FACS to investigate differences in the dissemination of infectious units in absence of aggregate induction. **(C) **Cell fusion is induced by wildtpe MVA but less so by MVA-CR19. Red immunofluorescence against MVA antigens serves as a positive control for infection. Blue fluorescence of DNA is shown for orientation. MVA-negative cells next to infected cells are shown in the panels where virus was added to a multiplicity of infection (MOI) of 0.1.

Motivated to discover new phenotypes, we quantified replication of successive MVA passages in aggregated CR suspension cultures. Because titers increased slightly within 10 passages, viral genomic DNA of early and late passages was sequenced. Of the advanced passage, a contiguous sequence of 135 kb was recovered and revealed a genotype (which we call MVA-CR) where the structural proteins A3L, A9L and A34R (in vaccinia virus nomenclature) each carry a single amino acid exchange (Figure 1A). The novel genotype appears to accumulate in our system but to completely remove traces of wildtype plaque purification was performed. The pure isolate (called MVA-CR19) was further characterized and compared to the wildtype.

## Results

The aggregate-based process was developed to facilitate cell-to-cell spread, which appears to be an important mechanism for vaccinia virus replication. Surprisingly, multiplication of MVA-CR19 appears to be efficient also in single-cell avian suspension cultures (Figure 1B) with increased infectious titers in the cell-free supernatant. Because of this qualitative difference between wildtype and MVA-CR19, we hypothesized that a smaller fraction of the MVA-CR isolate remains cell associated and that this capacity allows viruses of the novel genotype to spread also in single cell suspensions. As one test of our proposed explanation we repeated the passaging experiments in adherent cultures. No mutations in the three genes that distinguish MVA-CR were detected, suggesting that the contribution of host cell properties to the observed changes in the virus population recovered from the suspension process may be negligible.

However, the MVA-CR phenotype is evident also in adherent cells: compared to wildtype MVA, plaques formed by MVA-CR19 on CR cell monolayers in comet assays appear to be larger and to develop earlier [5]. These results are consistent with mechanisms that allow MVA-CR19 to replicate, infect or uncoat faster, or be released with greater efficiency from host cells. For further characterization of this effect, adherent cells were infected with a high multiplicity of 10 and briefly subjected to a pH shift. This is predicted to activate the viral fusion apparatus so that cell-associated viruses in a confluent cell monolayer can induce formation of syncitia [6]. As shown in Figure 1C, cell fusion appears to be less pronounced in cultures infected with MVA-CR suggesting that either fewer virions of this genotype remain cell associated or that fusion may be less important for entry of such virions.

A molecular basis for the proposed improved MVA-CR19 dissemination is that all three of the observed mutations each target a different component of the complex viral particles, the core and the different membranes of the mature intracellular and extracellular virions. We are in the process of generating various combinations of recombinant MVAs to determine whether all three factors need to cooperate to produce the observed effects or whether a single gain of function mutation in any one or two factors is sufficient.

## Conclusions

Compared to wildtype MVA, plaques formed by MVA-CR19 on adherent CR cells appear to be larger and to develop earlier. Titers are slightly higher in complete lysates and significantly elevated in cell-free supernatants. MVA-CR19 replicates efficiently without aggregate induction also in single cell suspension cultures. We hypothesize that a greater fraction of MVA-CR19 escapes the hosts for infection of distant targets. In such a model the new genotype should not confer a significant advantage to viruses spreading in cell monolayers, and indeed we could not generate the MVA-CR genotype by passaging in adherent cultures. Attenuation has yet to be confirmed for MVA-CR but host cell-restriction appears to have been fully maintained for Vero and HEK 293 cells.

Supply of an injectable vaccine preparation may be facilitated with this strain as production in single cell suspension using only a cell proliferation medium is less complex compared to the current protocol that requires cell aggregate induction by addition of a virus production medium. Furthermore, MVA-CR has a tendency to accumulate in the extracellular volume. Purification of live virus out of a cell-free suspension may allow enhanced purity compared to a process that initiates with a complete lysate containing the full burden of unwanted host cell-derived components.
